# Droplet-based single-cell RNA sequencing: decoding cellular heterogeneity for breakthroughs in cancer, reproduction, and beyond

**DOI:** 10.1186/s12967-025-06996-0

**Published:** 2025-10-14

**Authors:** Haoxuan Luo, Ansar Hussain, Musavir Abbas, Lun Yuan, Youfeng Shen, Ziyuan Zhang, Guorong Sun, Xiujuan Yin, Shan Huang

**Affiliations:** 1 Chongqing Medical and Pharmaceutical College, Chongqing, China; 2 Anhui Province Biomedical Sciences and Health Laboratory, First Affiliated Hospital of USTC, Hefei National Laboratory for Physical Sciences at Microscale, the CAS Key Laboratory of Innate Immunity and Chronic Disease, School of Basic Medical Sciences, Division of Life Sciences and Medicine, Division of Reproduction and Genetics, University of Science and Technology of China, Hefei, 230027 China; 3 Chongqing Precision Medical Industry Technology Research Institute, Chongqing, 400000 China

**Keywords:** Spatial multi-omics, Circulating tumor cells (CTCs), Epigenetic reprogramming, Microfluidic cost-reduction, Preimplantation genetic diagnosis (PGD), 10× Genomics chromium, GEMs, Tumor heterogeneity

## Abstract

Droplet-based single-cell RNA sequencing (scRNA-seq) has redefined biological research by resolving cellular heterogeneity with an unprecedented precision. This review synthesizes the technological evolution of the 10× Genomics Chromium platform, its Gel Bead-in-Emulsion (GEM)s technology, and end-to-end workflows, while critically addressing persistent challenges, such as cell capture variability (30–75% efficiency), barcode collisions (< 5% multiplet rates), and mRNA capture limitations (10–50% efficiency). We spotlight two transformative applications: (1) cancer research, where scRNA-seq unveils rare circulating tumor cells (CTCs) and dynamic tumor microenvironments, and (2) reproductive medicine, enabling breakthroughs in primordial germ cell (PGC) epigenetics and pre-implantation diagnostics. Unlike prior reviews, we systematically evaluated innovative solutions-including UMIs, computational demultiplexing, and microfluidic cost-reduction strategies (40–60% savings)-and propose a roadmap for integrating spatial transcriptomics and multi-omics approaches. Looking ahead, we identify three frontiers poised to redefine the field: (i) single-cell epigenome-transcriptome co-profiling, (ii) AI-driven analysis of multimodal datasets, and (iii) scalable microfluidics for clinical adoption. This review is of key significance in bridging cutting-edge technology with translational impact in oncology and reproductive biology, while outlining a roadmap for the evolution of the field.

**Graphical abstract:**

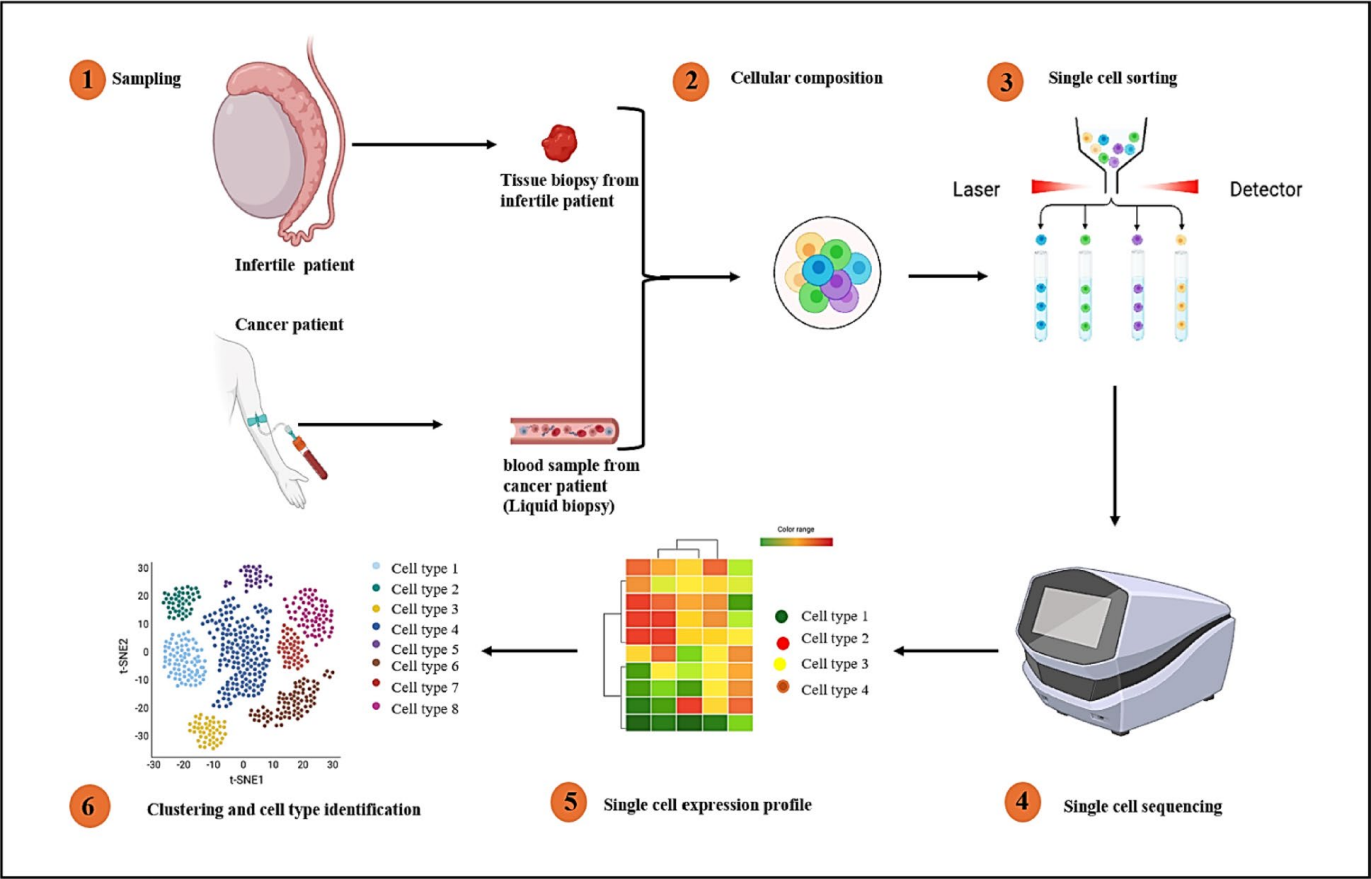

**Supplementary Information:**

The online version contains supplementary material available at 10.1186/s12967-025-06996-0.

## Introduction

The advent of single-cell RNA sequencing (scRNA-seq) has fundamentally altered our capacity to investigate cellular diversity, overcoming the limitations of bulk RNA sequencing, which obscures critical heterogeneity within biological systems [1–3]. While bulk methods provide population averages, they inevitably mask the nuanced differences between individual cells that drive development, disease progression, and tissue function. This resolution gap has been bridged by droplet-based scRNA-seq platforms, which leverage microfluidic partitioning to enable parallel transcriptomic analysis of thousands to millions of individual cells [4, 5]. While droplet-based platforms share core principles, their performance characteristics differ substantially (Supplementary Table 1). The 10× Genomics Chromium system-currently the gold standard—achieves superior cell capture efficiency (65–75% vs. 30–60% for alternatives) and gene detection sensitivity (1000–5000 genes/cell), albeit at higher per-cell costs (0.20–1.00). These advantages stem from its optimized microfluidics and barcoding chemistry, which also reduce multiplet rates to < 5% compared to 5–15% in Drop-seq [6, 7]. However, open platforms such as Drop-seq remain valuable for cost-sensitive studies, demonstrating how platform choice involves trade-offs between data quality and budget.

The power of this technology is exemplified by its core innovation, the Gel Bead-in-Emulsion (GEM) system, which combines barcoded oligonucleotides with nanoliter-scale droplets to uniquely label cellular mRNA [8, 9]. This elegant approach has enabled remarkable progress in just over a decade, from the first single-cell transcriptome protocol [10] to today’s high-throughput commercial systems [11]. Perhaps most significantly, these advances have catalyzed large-scale initiatives like the Human Cell Atlas [12], and transformed our understanding of cellular heterogeneity [13]. As the technology has matured, its applications have expanded dramatically. In cancer research, scRNA-seq has uncovered rare subclones and dynamic tumor microenvironments [14], while in reproductive biology, it has elucidated epigenetic reprogramming in primordial germ cells [15] and enhanced preimplantation genetic diagnosis [16]. These breakthroughs share a common foundation in the ability of the technology to profile thousands of cells simultaneously while maintaining single-cell resolution.

Despite these successes, challenges remain in mRNA capture efficiency (typically 10–50% of cellular transcripts) and ambient RNA contamination. Emerging integrations with spatial transcriptomics and multi-omics platforms promise to address these limitations [3, 17]. This review advances the field in the following ways:

- Providing the first side-by-side comparison of 10× Genomics versus open platforms (Drop-seq, inDrops) for clinical applications [6].
- Resolving controversies in oligo (dT) bias through template-switch oligo strategies. This limitation has been effectively addressed through template-switch oligo (TSO) strategies, which enable cDNA synthesis independent of poly(A) tails by binding to the 3′ end of newly synthesized cDNA during reverse transcription (Fig. 4).
- Linking scRNA-seq findings to actionable diagnostics (MALBAC for embryo screening).

## Droplet-based single-cell RNA sequencing (scRNA-seq): a detailed explanation

**Fig. 1:**
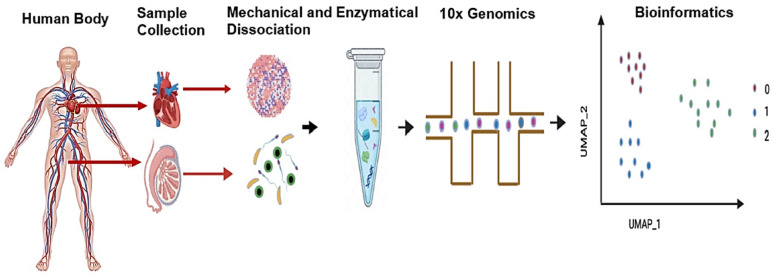
Overview of droplet-based single-cell RNA sequencing (scRNA-seq). Droplet-based scRNA-seq captures single cells and their RNA in tiny oil droplets, labeling each cell with a unique barcode for high-throughput analysis. Technologies such as 10× Genomics Chromium enable the parallel profiling of thousands of cells, revealing gene expression heterogeneity. It’s widely used in immunology, cancer, and developmental biology research

The emergence of droplet-based single-cell RNA sequencing (scRNA-seq) has fundamentally transformed our ability to study cellular heterogeneity at unprecedented resolution [4]. This technology overcomes the inherent limitations of bulk RNA sequencing by employing sophisticated microfluidic systems to isolate individual cells within nanoliter-scale droplets, thereby creating discrete reaction chambers for parallel transcriptome analysis [18]. The core innovation lies in the integration of barcoded gel beads within a water-in-oil emulsion system, where each bead carries millions of oligonucleotides designed for specific mRNA capture and molecular labeling [8] (Fig. 1).

**Fig. 2:**
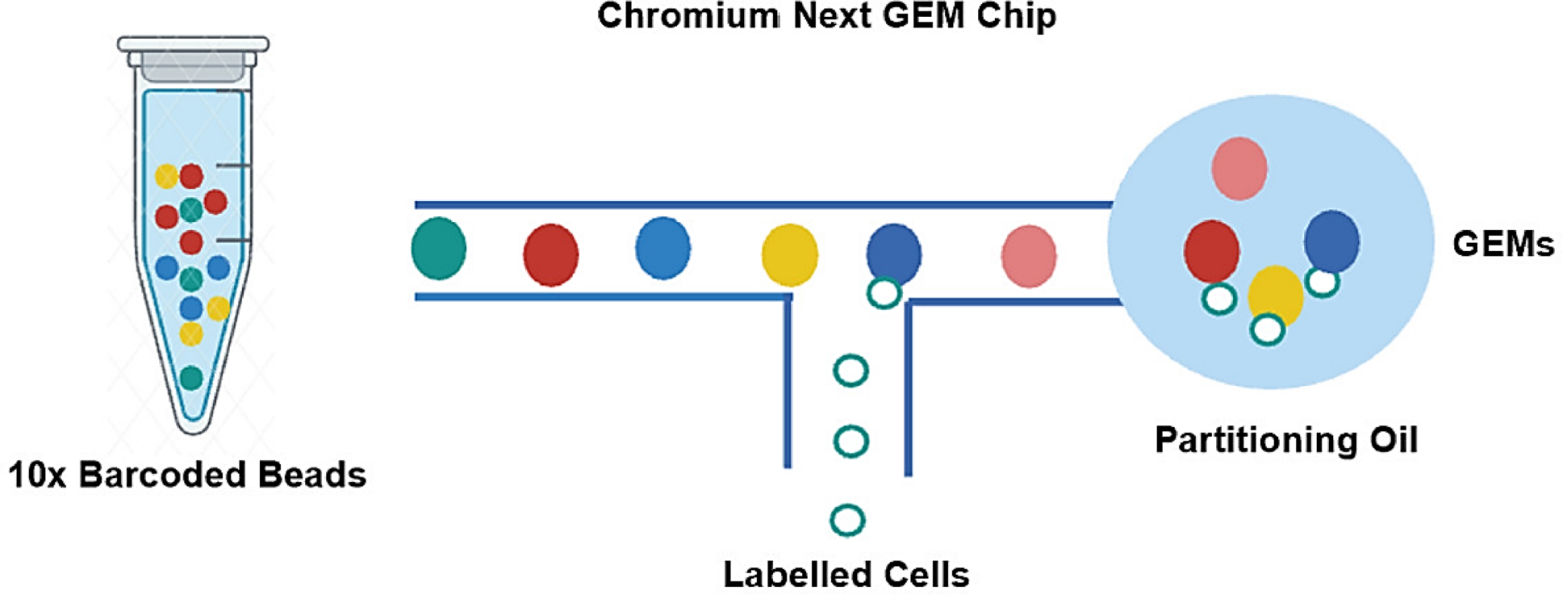
GEMs (gel bead-in-emulsion) formation. GEMs (gel bead-in-emulsions) are formed by combining barcoded single cell VDJ 5′ gel beads, a master mix containing cells and reaction reagents, and partitioning oil within a Chromium Next GEM Chip K. The microfluidic system generates nanoliter-scale droplets where each GEM encapsulates a single cell and gel bead, enabling cell-specific barcoding of nucleic acids through the released oligonucleotides

The methodological workflow begins with the careful preparation of a high-quality single-cell suspension, requiring optimization of both cell concentration (typically 700–1200 cells/μL) and viability (> 85%) [19]. As this suspension passes through precisely engineered microfluidic channels, it merges with barcoded beads and partitions oil to generate monodisperse droplets [20]. Within each droplet, cell lysis releases mRNA that binds to the bead's oligo (dT) primers, followed by reverse transcription to produce cDNA molecules tagged with unique cellular identifiers [9]. This elegant barcoding strategy enables subsequent computational deconvolution of pooled sequencing data while accounting for amplification biases through molecular counting [21] (Fig. 2).

**Fig. 3:**
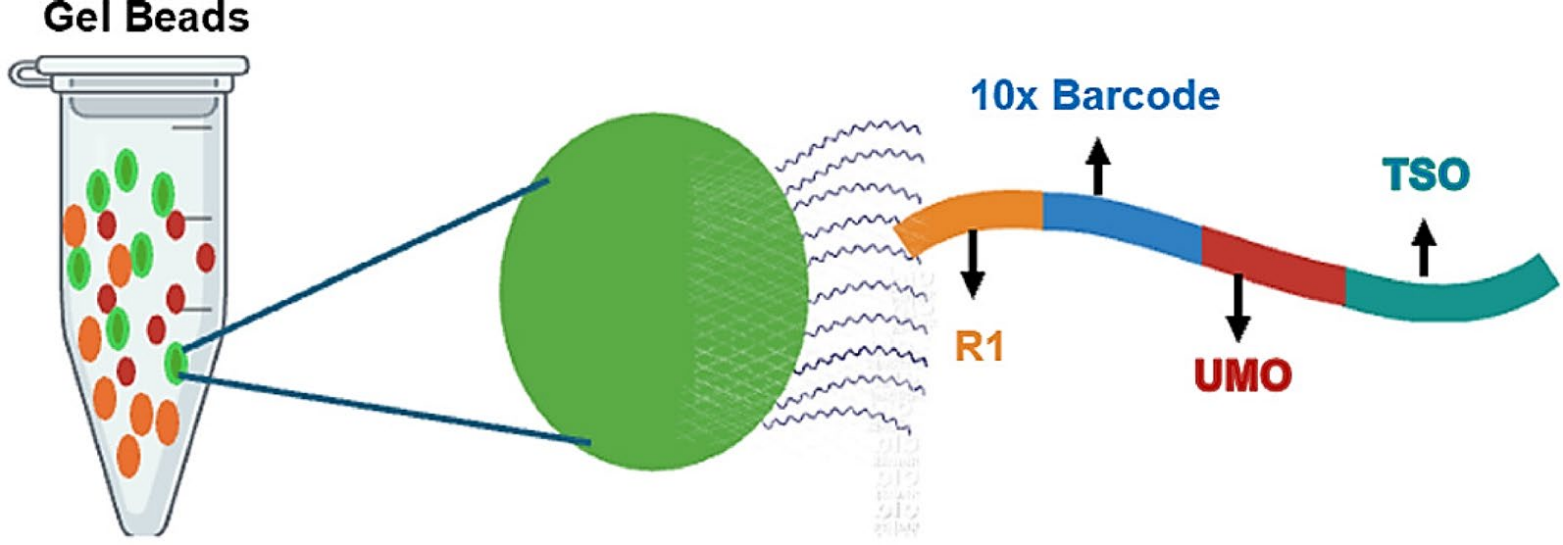
Barcoded beads for mRNA capture and quantification. Each bead contains millions of barcoded oligos with poly(dT) for mRNA capture. These include cell barcodes and UMIs for single-cell resolution and quantification. Upon dissolution, they tag transcripts efficiently within each GEM

Several key features distinguish droplet-based methods from alternative single-cell approaches. First, their remarkable throughput capacity allows processing of thousands to millions of cells in a single experiment, with per-cell costs decreasing substantially at scale. Second, the gentle encapsulation process better preserves cell integrity compared to some plate-based methods. Third, the standardized workflow minimizes technical variability between experiments [22]. Recent advancements have further expanded the technology's capabilities through integration with protein detection methods (CITE-seq), chromatin accessibility profiling (ASAP-seq), and compatibility with fixed or frozen samples [23]. The barcoded bead structure, which enables mRNA capture and unique molecular identifier (UMI) labeling, is illustrated in Fig. 3.

**Fig. 4:**
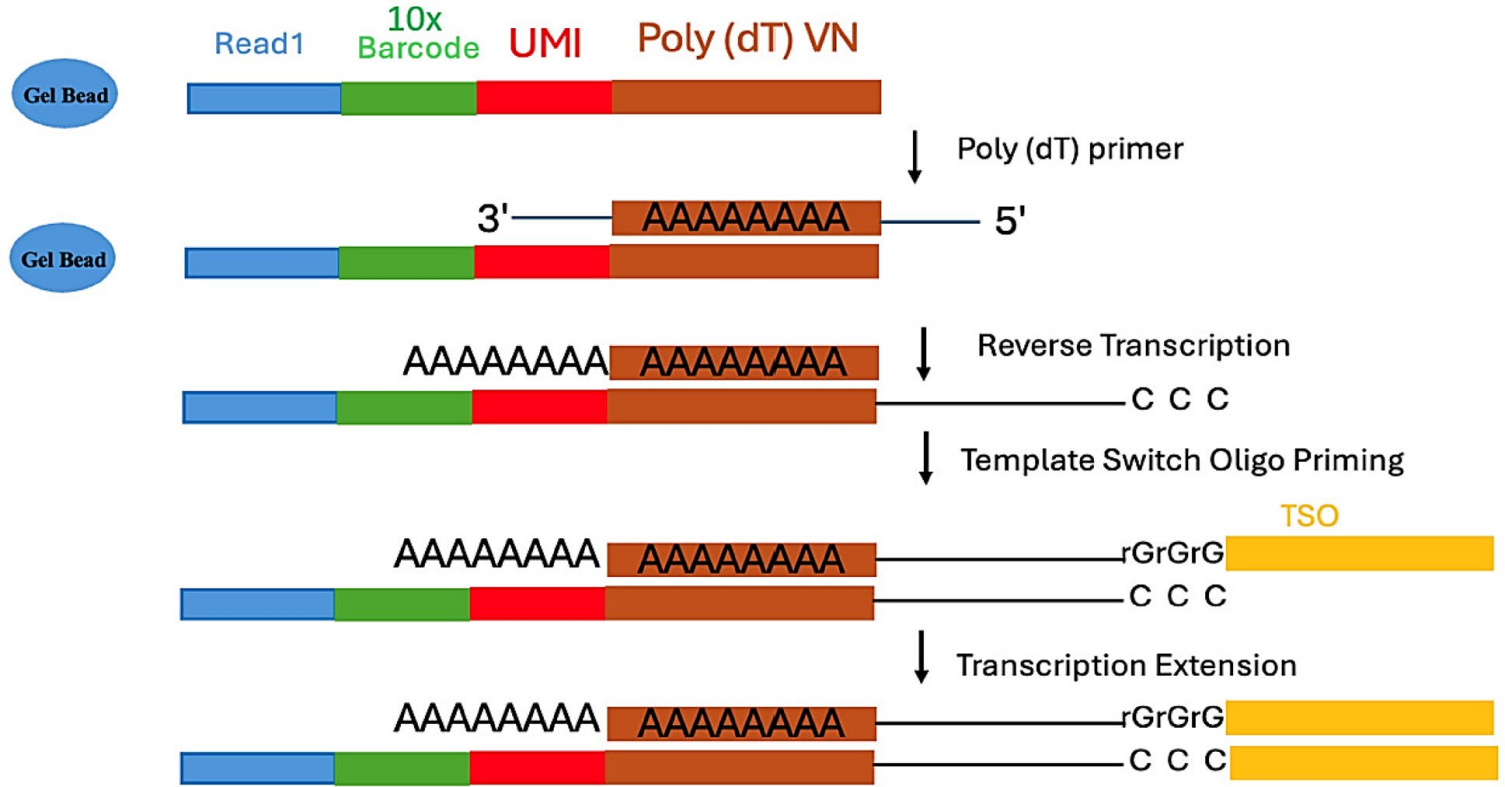
cDNA synthesis and barcoding in partitioned GEMs. After GEM formation, the Gel Bead dissolves to release oligonucleotides while simultaneously lysing any encapsulated cells. The released oligonucleotides contain key functional elements including an Illumina R1 sequencing primer, a 16-nucleotide 10× Barcode for sample identification, a unique molecular identifier (UMI) for molecule counting, and a template switch oligo (TSO) sequence. These components combine with the cell lysate and a Master Mix containing reverse transcription reagents and poly(dT) primers. During incubation, this system produces barcoded full-length cDNA copies of all polyadenylated mRNAs within each GEM

The technical performance of droplet-based systems is characterized by several key metrics. Typical experiments achieve capture of 500–5000 genes per cell, with UMI counts ranging from 1000 to 50,000 molecules per cell [24]. The cDNA synthesis process within partitioned GEMs, including reverse transcription and barcoding, is detailed in Fig. 4. The multiplet rate remains below 5% when following optimal loading concentrations, while barcode collision probabilities are typically maintained at < 0.1% [7]. Recent protocol enhancements have improved mRNA capture efficiency to 10–50% of cellular transcripts and reduced ambient RNA contamination by 30–50%.

The impact of this technology is evident in its widespread adoption across diverse research applications. In cell atlas projects, it enables comprehensive tissue profiling at single-cell resolution. Cancer researchers employ it to dissect tumor microenvironments and identify rare subclones [25]. Immunologists leverage its ability to pair transcriptional profiles with immune receptor sequences, while developmental biologists use it to reconstruct lineage trajectories with remarkable precision [26].

As the field progresses, several promising directions are emerging. Microfluidic innovations aim to increase partitioning density and reduce droplet volumes. Molecular biology improvements focus on enhancing full-length transcript recovery and expanding omics data integration. Computational advances are addressing the challenges of demultiplexing, cell type annotation, and multimodal data integration. These developments, combined with decreasing costs and increasing automation, are making single-cell resolution analysis increasingly accessible to research institutions [27]. Several best practices are recommended for researchers implementing this technology. A careful experimental design should include power calculations that account for expected cellular heterogeneity, appropriate spike-in controls, and sufficient technical replication. Systematic quality control should monitor key metrics, including cell viability, doublet rates, and sequencing saturation. Troubleshooting guides are available to address common issues, such as low cell recovery or poor cDNA yield [28]. This comprehensive examination of droplet-based scRNA-seq technology highlights its current capabilities and future potential. As the methodology continues to evolve, it promises to further transform our understanding of cellular biology and accelerate progress in precision medicine [29]. The integration of continuous technical improvements with expanding biological applications ensures that droplet-based single-cell approaches will remain at the forefront of genomic research for the foreseeable future [30].

## Applications

Droplet-based single-cell RNA sequencing is a powerful tool across biological and medical research [22]. Cell atlas projects, such as the Human Cell Atlas, enable detailed mapping of cellular diversity. In cancer research, it helps identify resistant subclones and tumor microenvironment dynamics [12]. Immunology studies benefit from linking transcriptional profiles to immune receptor sequences, advancing our understanding of adaptive responses [31]. Developmental biology leverages this technology to reconstruct lineage trajectories and cell fate decisions in various organisms. In addition, it impacts neuroscience, infectious diseases, regenerative medicine, and drug discovery, offering unprecedented single-cell resolution for exploring biological systems [13].

### Application of single-cell RNA sequencing in cancer

Single-cell RNA sequencing (scRNA-seq) has emerged as a revolutionary approach in cancer research, providing unprecedented resolution for analyzing tumor biology at the cellular level [32]. This technology has fundamentally transformed our understanding of tumor heterogeneity, metastatic progression, and therapeutic resistance mechanisms [33]. By enabling transcriptomic profiling of individual cells, scRNA-seq has revealed complex cellular ecosystems within tumors that were previously obscured by bulk sequencing methods [34].

**Fig. 5:**
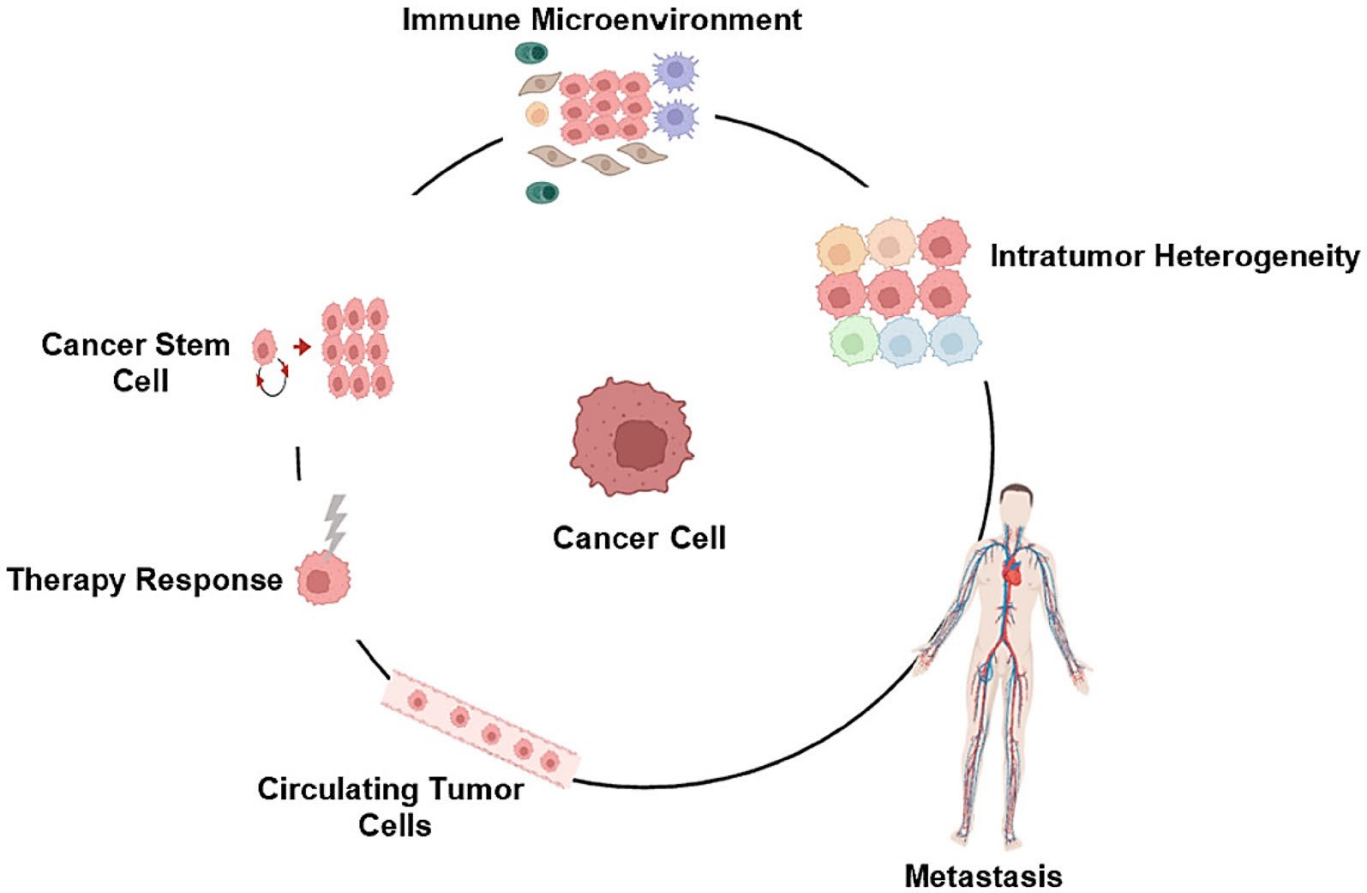
scRNA-seq reveals rare CTC subpopulations (red) undetectable by bulk sequencing. Current gold-standard scRNA-seq protocols predominantly utilize oligo(dT) primers for reverse transcription, which selectively capture polyadenylated mRNA transcripts. While effective for protein-coding genes, this approach systematically excludes important regulatory RNAs lacking poly(A) tails, including many long non-coding RNAs and microRNAs

In oncology applications, scRNA-seq has provided groundbreaking insights into tumor biology [35]. This technology has proven particularly valuable for identifying rare drug-resistant subpopulations, characterizing complex tumor microenvironment interactions, and analyzing circulating tumor cells (Fig. 5) [36]. However, significant technical hurdles remain, particularly in the isolation of circulating tumor cells where capture efficiency varies dramatically (0.004–69.5%) depending on the specific markers and methods employed [37]. Furthermore, data interpretation is frequently complicated by contamination from normal blood cells and technical artifacts introduced during sample processing [38].

The future of scRNA-seq in cancer research lies in its integration with complementary single-cell omics technologies [32]. Combining transcriptomic data with information about DNA methylation patterns, chromatin accessibility, and protein expression promises to provide more comprehensive understanding of tumor biology [39]. Spatial transcriptomic methods are particularly promising as they bridge the critical gap between single-cell resolution and tissue context [3]. Concurrent advances in computational biology have improved our ability to distinguish true biological signals from technical artifacts, enabling more robust data interpretation [40].

### Application of single-cell RNA sequencing in the field of reproductive medicine

Single-cell sequencing has revolutionized our understanding of reproductive biology by enabling high-resolution analysis of cellular heterogeneity and molecular dynamics during gametogenesis and early development [15]. This approach is particularly valuable for studying human primordial germ cells (PGCs), which are challenging to obtain and exhibit complex epigenetic reprogramming. Recent single-cell transcriptomic and methylome analyses of human PGCs across developmental stages have revealed several critical findings that distinguish them from murine models [41].

While human PGCs share expression of pluripotency markers OCT4, NANOG, and REX1 with mouse PGCs, they notably lack SOX2 expression, instead expressing SOX15 and SOX17 [42]. These cells demonstrate significant transcriptional heterogeneity during meiotic entry, likely reflecting asynchronous progression through meiosis [42]. A key epigenetic event in female PGCs is the reactivation of the randomly inactivated X chromosome, which occurs by 4 weeks of development [43]. Perhaps most strikingly, human PGCs undergo dramatic genome-wide DNA demethylation, decreasing from 92% methylation in post implantation embryos to just 7% by 10–11 weeks gestation—the lowest methylation level observed in any normal human cell type [44]. Interestingly, while most functional genomic elements become completely demethylated, certain repeat elements maintain residual methylation, suggesting potential mechanisms for transgenerational epigenetic inheritance [45]. The preservation of stable gene expression patterns despite massive DNA demethylation implies compensatory roles for other epigenetic regulators, particularly histone modifications [46].

**Fig. 6:**
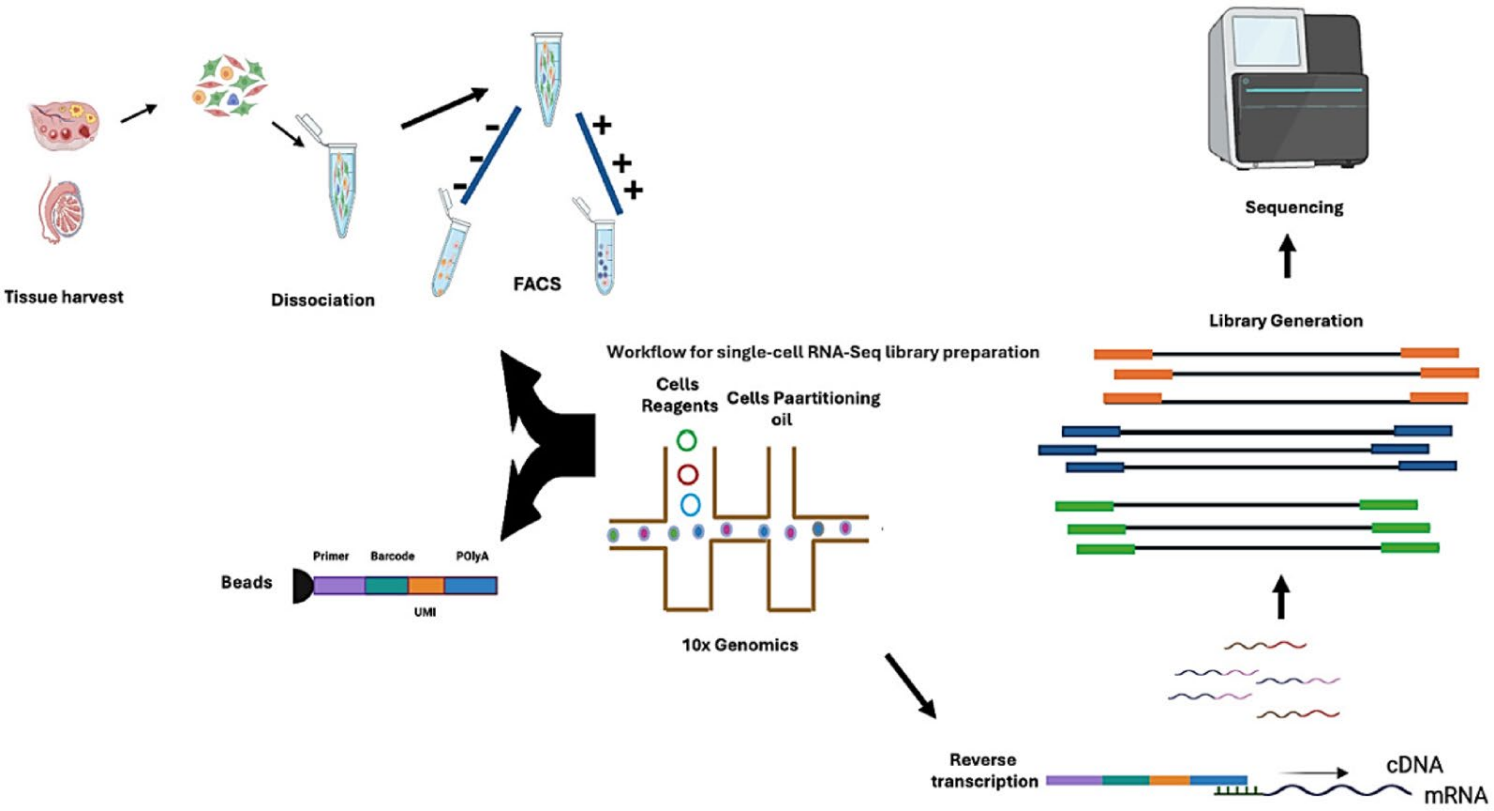
Schematic overview of scRNA-seq in gonadal tissues. Gonadal tissues are enzymatically dissociated, and single-cell suspensions are purified via FACS or MACS. For microfluidics-based scRNA-seq, individual cells are paired with uniquely barcoded beads. Following cell lysis, mRNA is captured, reverse-transcribed, and converted into sequencing libraries containing bead-specific barcodes and UMIs for accurate transcript quantification

Single-cell bisulfite sequencing approaches, including scRRBS and WGBS, have provided unprecedented views of DNA methylation dynamics in early human embryos [47]. The workflow for gonadal tissue analysis is shown in Fig. 6. These studies revealed that the major wave of genome-wide demethylation completes by the 2-cell stage, with paternal genome demethylation occurring more rapidly than maternal genome reprogramming [48]. By the zygotic stage, male pronuclei exhibit lower methylation levels than female pronuclei [49]. Complementary single-cell RNA-seq analyses have generated comprehensive transcriptional maps of human preimplantation development, identifying both known and novel lncRNAs, including maternally expressed species [50].

These fundamental discoveries have important clinical applications in reproductive medicine. The MALBAC method has been successfully adapted for preimplantation genetic diagnosis, enabling detection of both monogenic disorders and chromosomal abnormalities [51]. The MARSALA approach, combining next-generation sequencing with MALBAC, provides single-molecule resolution for embryo screening, significantly improving diagnostic accuracy by reducing false-positive and false-negative results [16]. These technological advances not only enhance the safety of assisted reproductive technologies but also provide critical insights into epigenetic regulation during human development, with implications for understanding and treating germ cell abnormalities and epigenetic diseases.

### Single cell RNA sequencing in kidney disease

Single-cell RNA sequencing (scRNA-seq) has revolutionized our understanding of kidney disease pathogenesis by revealing cellular trajectories, therapeutic targets, and diagnostic biomarkers across major renal conditions. In lupus nephritis (LN), scRNA-seq has identified type I IFN-response gene upregulation in tubular cells and keratinocytes, suggesting that skin biopsies may be potential biomarkers [52, 53]. Immune cell profiling has demonstrated the utility of urine as a non-invasive surrogate for kidney biopsies [54]. For renal cell carcinoma (RCC), studies uncovered tumor microenvironment heterogeneity, including immune cell infiltration patterns in clear-cell RCC [55] and metastatic RCC subpopulations responsive to combination therapies [56]. In diabetic nephropathy (DN), scRNA-seq revealed altered glomerular-tubular crosstalk via integrin pathways and identified FGF1 as a renoprotective factor [57]. Acute kidney injury (AKI) research leveraged scRNA-seq to map injury-specific cell states like failed-repair proximal tubule cells (Kirita et al. 2020) and COVID-19-associated tubular injury pathways [57]. IgA nephropathy studies highlighted dysregulated JCHAIN expression in mesangial cells and CD8+ T cell dysfunction [58, 59]. Collectively, these advances demonstrate the transformative role of scRNA-seq in elucidating disease mechanisms, refining diagnostics, and guiding personalized therapies in nephrology.

## Challenges and solutions

While technical noise and amplification biases remain challenges, [6] recent innovations mitigate these limitations:

- UMIs reduce PCR duplicates, improving quantification accuracy [60].
- Microfluidic optimizations (FIDELITY system) cut droplet instability by 30% [61].
- Computational tools (SoupX) remove ambient RNA contamination [60].

Multiple factors contribute to this challenge, including variability in cell isolation procedures, stochastic gene expression patterns characteristic of single cells, and amplification biases introduced during library preparation [62]. While the use of synthetic spike-in controls and standardized protocols has improved experimental reproducibility, the field continues to lack robust biological reference standards that adequately represent the complexity of single-cell transcriptomes [63] Oil droplet-based single-cell sequencing methods, including Drop-seq, inDrops, and 10× Genomics platforms, have revolutionized single-cell analysis by enabling high-throughput profiling of thousands of cells [22]. However, these methods face several technical challenges that can impact data quality and experimental outcomes [64]. One major limitation is the inconsistent cell encapsulation efficiency, where droplets may contain no, single, or multiple cells [65]. This issue is particularly problematic for rare cell populations or large cells that are difficult to encapsulate [66]. Optimizing cell concentration and microfluidic parameters can help mitigate this problem, but complete elimination of multiplets remains challenging [30].

Barcode-related issues present another significant challenge in droplet-based methods. Barcode collisions occur when multiple cells share the same barcode due to insufficient barcode diversity or bead aggregation [7]. Additionally, ambient RNA from lysed cells can contaminate droplets, leading to false signals in the data [63]. These problems can be partially addressed by using barcode libraries with greater complexity and implementing computational methods for doublet detection and ambient RNA removal [66]. However, these solutions may not completely eliminate all artifacts, particularly in complex samples with high cellular heterogeneity [67].

The amplification process in droplet-based sequencing introduces its own set of challenges [68]. PCR amplification bias favors highly expressed genes, resulting in uneven transcript coverage and potential dropout of low-abundance transcripts [69]. This technical noise can distort gene expression measurements and complicate downstream analysis [70]. While molecular techniques like UMI-based normalization and improved reverse transcription protocols have helped reduce these biases, they cannot completely eliminate the stochastic nature of single-cell RNA amplification [60]. These limitations become particularly apparent when studying subtle transcriptional differences between cell states [64]. Droplet stability represents another critical factor affecting data quality [62]. The physical properties of the emulsion system, including surfactant composition and microfluidic conditions, must be carefully controlled to prevent droplet merging or rupture [71]. Even minor deviations from optimal conditions can lead to cell loss or barcode mixing, potentially compromising entire experiments [72]. While advances in microfluidics and surfactant chemistry have improved droplet stability, these systems remain sensitive to environmental factors and require precise calibration [30].

From a practical standpoint, the high cost and limited scalability of commercial droplet-based systems present barriers to widespread adoption, particularly for large-scale studies [30]. While open-source alternatives and sample multiplexing strategies have helped reduce costs, throughput limitations persist for studies requiring analysis of hundreds of thousands to millions of cells [66]. These challenges highlight the need for continued innovation in single-cell technologies to improve accessibility and data quality while reducing costs. Future developments may combine the strengths of droplet-based methods with emerging technologies to overcome these limitations-such as UDA-seq, a universal workflow that integrates a post-indexing step to enhance throughput and systematically adapt existing droplet-based single-cell multimodal methods. UDA-seq is benchmarked across various tissues and cell types, enabling several common multimodal analyses, including single-cell co-assay of RNA and VDJ, RNA and chromatin, and RNA and CRISPR perturbation. Notably, UDA-seq facilitated the efficient generation of over 100,000 high-quality single-cell datasets from three dozen frozen clinical biopsy specimens within a single-channel droplet microfluidics experiment [73]. However, the robustness of this approach in identifying rare cell subpopulations associated with clinical phenotypes and exploring the vulnerability of cancer cells needs further validation. Such innovations could enable more robust single-cell analyses while improving scalability and cost-efficiency.

## Conclusion

Droplet-based single-cell RNA sequencing (scRNA-seq) has revolutionized our understanding of cellular heterogeneity, offering unprecedented resolution in deciphering complex biological systems. This review highlights the transformative impact of 10× Genomics Chromium technology, particularly its GEM-based workflow, in advancing cancer research (rare CTC detection and tumor microenvironment mapping) and reproductive medicine (e.g., epigenetic reprogramming in PGCs and preimplantation diagnostics). Despite persistent challenges-such as mRNA capture efficiency (10–50%) and barcode collisions (< 5% multiplet rates)-innovations like UMIs, TSO strategies, and computational tools (SoupX) have significantly improved data accuracy and reproducibility. Looking ahead, the integration of spatial multi-omics, AI-driven analysis, and scalable microfluidics (UDA-seq) promises to further democratize single-cell technologies, enhance clinical translation, and unlock new frontiers in precision medicine. By bridging technological advancements with biological discovery, scRNA-seq remains indispensable for unraveling developmental, pathological, and therapeutic mechanisms at single-cell resolution.

## Supplementary Information

Below is the link to the electronic supplementary material.

Supplementary Material 1.

## Data Availability

The authors confirm that all the data already presented in the article.

## Abbreviations

scRNA-seq: Single-cell RNA sequencing
GEM: Gel bead-in-emulsion (core technology of 10× Genomics)
UMI: Unique molecular identifier (for reducing PCR amplification biases)
CITE-seq: Cellular indexing of transcriptomes and epitopes by sequencing (combines RNA and protein detection)
ASAP-seq: ATAC-seq with select antigen profiling by sequencing (integrates chromatin accessibility and protein data)
MALBAC: Multiple annealing and looping-based amplification cycles (used in preimplantation genetics)
MARSALA: Mutated allele revealed by sequencing with aneuploidy and linkage analyses (embryo screening method)
CTC: Circulating tumor cell (studied via scRNA-seq in cancer)
PGC: Primordial germ cell (key focus in reproductive medicine)
hTERT: Human telomerase reverse transcriptase (mentioned in immunotherapy context)
LN: Lupus nephritis (kidney disease studied via scRNA-seq)
RCC: Renal cell carcinoma (cancer type analyzed with scRNA-seq)
DN: Diabetic nephropathy (kidney disease application)
AKI: Acute kidney injury (studied via scRNA-seq)
IgA: Immunoglobulin A (related to IgA nephropathy studies)
lncRNA: Long non-coding RNA (highlighted in reproductive studies)
miRNA: MicroRNA (excluded by oligo(dT) primers in scRNA-seq)
scRRBS: Single-cell reduced representation bisulfite sequencing (for DNA methylation)
WGBS: Whole-genome bisulfite sequencing (methylation analysis)
XCI: X chromosome inactivation (mentioned in germ cell studies)
SNP: Single nucleotide polymorphism
FACS: Fluorescence-activated cell sorting (for cell isolation)
MACS: Magnetic-activated cell sorting (cell purification method)
TSO: Template switch oligo (used in cDNA synthesis)
R1: Read 1 (Illumina sequencing primer)
IFN: Interferon (mentioned in lupus nephritis studies)
FGF1: Fibroblast growth factor 1 (renoprotective factor in kidney disease)
