# Supplementary material for: Droplet-based single-cell RNA sequencing: decoding cellular heterogeneity for breakthroughs in cancer, reproduction, and beyond
Source: J Transl Med. 2025 Oct 14;23:1091. doi: 10.1186/s12967-025-06996-0 (PMC12522672; doi:10.1186/s12967-025-06996-0)
Supplement: Supplementary file 1 — Supplementary Material 1. [file 12967_2025_6996_MOESM1_ESM.docx]

| **Features** | **10x Genomics**  **Chromium** | **Drop-Seq** | **inDrops** | **References** |
| --- | --- | --- | --- | --- |
| Cell capture efficiency | 65-75% | 30-60% | 40-60% | (Chen, Ning, & Shi, 2019; Matuła, Rivello, & Huck, 2020) |
| Gene detection (genes/cell) | 1,000-5,000 | 500-3,000 | 500-3,000 | (Lareau, Ma, Duarte, & Buenrostro, 2020; Zhang et al., 2022) |
| Multiple rate | <5% | 5-15% | ~10% | (Lareau et al., 2020; Moragues et al., 2023) |
| Barcode collision rate | <0.1% | Up to 1% | ~0.5% | (Lareau et al., 2020) |
| UMI support | Yes | Yes | Yes | (Salomon et al., 2019) |
| Cost per cell | $0.20-1.00 | <$0.10 | ~$0.10 | (Chen et al., 2019; Moragues et al., 2023) |
| Open-source availability | No | Yes | Yes | (Salomon et al., 2019) |
| Commercial support | Yes | No | No | (Moragues et al., 2023; Salomon et al., 2019) |

**Supplementary Table 1 – Comparison of Droplet-Based scRNA-seq Platforms**

**References**

Chen, G., Ning, B., & Shi, T. (2019). Single-cell RNA-seq technologies and related computational data analysis. *Frontiers in Genetics, 10*, 317.

Lareau, C. A., Ma, S., Duarte, F. M., & Buenrostro, J. D. (2020). Inference and effects of barcode multiplets in droplet-based single-cell assays. *Nature communications, 11*(1), 866.

Matuła, K., Rivello, F., & Huck, W. T. (2020). Single‐cell analysis using droplet microfluidics. *Advanced Biosystems, 4*(1), 1900188.

Moragues, T., Arguijo, D., Beneyton, T., Modavi, C., Simutis, K., Abate, A. R., . . . Griffiths, A. D. (2023). Droplet-based microfluidics. *Nature Reviews Methods Primers, 3*(1), 32.

Salomon, R., Kaczorowski, D., Valdes-Mora, F., Nordon, R. E., Neild, A., Farbehi, N., . . . Gallego-Ortega, D. (2019). Droplet-based single cell RNAseq tools: a practical guide. *Lab on a Chip, 19*(10), 1706-1727.

Zhang, H., Huang, C., Li, Y., Gupte, R., Samuel, R., Dai, J., . . . Han, A. (2022). FIDELITY: A quality control system for droplet microfluidics. *Science Advances, 8*(27), eabc9108.
